# The Relationship between Asthma and Depression in Primary Care Patients: A Historical Cohort and Nested Case Control Study

**DOI:** 10.1371/journal.pone.0020750

**Published:** 2011-06-15

**Authors:** Paul Walters, Peter Schofield, Louise Howard, Mark Ashworth, André Tylee

**Affiliations:** 1 Health Service and Population Research Department, Institute of Psychiatry, King's College London, London, United Kingdom; 2 Division of Health and Social Care Research, Department of Primary Care and Public Health Sciences, King's College London, London, United Kingdom; University of Oxford, United Kingdom

## Abstract

**Background and Objectives:**

Asthma and depression are common health problems in primary care. Evidence of a relationship between asthma and depression is conflicting. Objectives: to determine 1. The incidence rate and incidence rate ratio of depression in primary care patients with asthma compared to those without asthma, and 2. The standardized mortality ratio of depressed compared to non-depressed patients with asthma.

**Methods:**

A historical cohort and nested case control study using data derived from the United Kingdom General Practice Research Database. Participants: 11,275 incident cases of asthma recorded between 1/1/95 and 31/12/96 age, sex and practice matched with non-cases from the database (ratio 1∶1) and followed up through the database for 10 years. 1,660 cases were matched by date of asthma diagnosis with 1,660 controls. Main outcome measures: number of cases diagnosed with depression, the number of deaths over the study period.

**Results:**

The rate of depression in patients with asthma was 22.4/1,000 person years and without asthma 13.8 /1,000 person years. The incident rate ratio (adjusted for age, sex, practice, diabetes, cardiovascular disease, cerebrovascular disease, smoking) was 1.59 (95% CI 1.48–1.71). The increased rate of depression was not associated with asthma severity or oral corticosteroid use. It was associated with the number of consultations (odds ratio per visit 1.09; 95% CI 1.07–1.11). The age and sex adjusted standardized mortality ratio for depressed patients with asthma was 1.87 (95% CI: 1.54–2.27).

**Conclusions:**

Asthma is associated with depression. This was not related to asthma severity or oral corticosteroid use but was related to service use. This suggests that a diagnosis of depression is related to health seeking behavior in patients with asthma. There is an increased mortality rate in depressed patients with asthma. The cause of this needs further exploration. Consideration should be given to case-finding for depression in this population.

## Introduction

After hypertension asthma is the most common chronic illness in primary care in the United Kingdom with a prevalence 6% [1]. Depression also has a high prevalence in primary care of between 5 and 10% [2]. Chronic physical health problems are reported to be associated with increased rates of depression [3].

Whether there is an association between asthma and depression is unclear. In secondary care populations up to 50% of patients with asthma have been reported to have clinically significant depressive symptoms and over a third of asthmatic outpatients have been found to have a major depressive episode [4], [5], [6], [7], [8], [9]. The World Mental Health Survey found an age and sex adjusted odds ratio of 1.6 for depression in people with asthma compared to people without asthma [10], [11]. However other researchers have failed to find an association [12], [13], [14], and there has been little research in primary care populations where the majority of people with these conditions are treated. Most studies to date have been cross-sectional in design and so the ability to explore potential associations has been limited. Longitudinal studies are needed to explore potential associations further.

The aims of this study were to determine the incidence of depression in primary care patients with asthma and the incidence rate ratio (IRR) of depression in this population compared to the general primary care population without asthma. We also wanted to explore potential mediators of this relationship and examine all cause mortality in patients with asthma and depression compared with non depressed patients with asthma.

We hypothesised that:

1. Primary care patients with asthma would have an increased incidence of depression compared with primary care patients without asthma after adjusting for age, sex , social deprivation, other common chronic medical conditions (coronary heart disease, diabetes and cardiovascular accidents) and oral corticosteroid medication use and smoking status.
2. Primary care patients with asthma and depression would have a higher age and sex standardized mortality rate compared with primary care patients with asthma but no depression.

## Methods

### Study design

This was a historical cohort study with a nested case-control study using data derived from the General Practice Research Database.

### General Practice Research Database

The UK General Practice Research Database (GPRD) is the world's largest database of anonymous longitudinal medical data from primary care (www.gprd.com). The database consists of the medical records of approximately 13 million primary care patients with 46 million years of validated data. In 1996, 480 practices across the UK contributed to the GPRD. Recorded data include diagnoses, clinical events, specialist referrals, prescription details, hospital admissions and outcomes. Each patient has a unique identifier which allows data held in 4 separate data-sets to be linked. The GPRD uses Oxford Medical Information System (OXMIS) codes and Read codes to store diagnostic information [15]. These are cross- referenced to the International Classification of Diseases (ICD9 and ICD10) by the UK National Health Service Information Authority.

The database is owned and managed by the Medicines and Healthcare Products Regulatory Agency (MHRA) in the UK. The quality of the data are regularly audited by the Office for National Statistics and only practices that are ‘up-to-research standard’ (UTS) are eligible to participate. The GPRD has been validated for use in respiratory epidemiology [16], and the results from the database for asthma are consistent with published studies on the epidemiology of asthma [17]. Comparisons of age and sex distributions are similar to those found in the National Population Census and the geographical distribution of practices participating in the GPRD is representative of the UK population [18].

### Historical cohort study

All patients aged 16 years or over with an incident diagnosis of asthma in their primary care record given between 1^st^ January 1995 and 31^st^ December 1996 and who had at least 24 months of UTS data before the start of the study window were identified from the GPRD, and followed up until 31 December 2006. A medical diagnosis of asthma was defined by a Read/ OXMIS code for asthma (codes available from authors). Read/OXMIS codes for asthma can de cross-referenced to ICD-10 asthma codes.

All cases with a recorded medical diagnosis of depression (as defined by Read/OXMIS codes) or depressive symptoms before the 1^st^ January 1995 were excluded from the cohort. Additionally, cases with a recorded diagnosis of schizophrenia or bipolar affective disorder (as defined by Read/OXMIS codes) over the study period were also excluded (Read/OXMIS codes used available from authors).

Patients were age (±2 years), sex and practice matched (ratio 1∶1) with patients who had not received a diagnosis of asthma over the same study period taken from the same GPRD base population of registered patients. GP practice was used as a proxy to control for socio-economic status as this was not directly available from data in the GPRD. GP practice has been found to correlate with socioeconomic status in the United Kingdom with practice postcode based Index of Multiple Deprivation (IMD) scores being correlated with population weighted IMD scores [19]. All patients had at least 24 months of UTS data prior to the index date of the case.

Patients were censored if they received a diagnosis of depression during the study period to prevent over-estimating any association due to recurrent depressive disorders in those that had already been diagnosed with depression during the study period.

### Nested case control study

Cases were defined as patients from the cohort study with a diagnosis of asthma and depression (as defined by Read/OXMIS codes) during the study period. Controls were defined as patients from the cohort study with a medical diagnosis of asthma but without a diagnosis of depression (as defined by Read/OXMIS codes) during the study period. Cases were matched to controls (ratio 1∶1) according to the date of asthma diagnosis (±1 month). The same inclusion and exclusion criteria were applied to both cases and controls and were the same as those for the cohort study.

### Measures

The exposure of interest was a GP recorded diagnosis of asthma. The outcome of interest was a GP recorded diagnosis of depression. Data on smoking status (defined as ever having smoked), co-morbidity with other common chronic illnesses (diabetes mellitus, cerebrovascular disease, coronary heart disease and congestive heart failure), anxiety disorders, asthma medications use (β-agonist use, inhaled corticosteroids and oral corticosteroids), and the number of GP consultations were obtained from the electronic medical records. The co-morbid illnesses were defined by a Read/OXMIS code for any of these diagnoses prior to the onset of depression in the cases, and prior to the same date in the matched control.

As has been validated in another study, asthma medications patients had received in the year before being given a diagnosis of depression were used as a proxy for asthma severity as follows: 1. Un-medicated asthma (no prescriptions) 2. Asthma medicated with at least one prescription of a short acting β-agonist. 3. Asthma medicated with at least one prescription for an inhaled corticosteroid with or without a long acting β-agonist. 4. asthma medicated with an oral corticosteroid [20]. The number of GP consultations in the year before the onset of depression for the cases and the number in the year before the same date for each matched control were recorded. These were further categorised into low use (<5 consultations), medium use (5–10 consultations), high use (10–19 consultations), and very high use (≥20 consultations).

### Statistical analysis

All data were analysed using STATA version 9 (STATA Corp, Texas). The incidence rate and summary rates of depression stratified by age and sex were calculated. Rate ratios and 95% confidence intervals were calculated for the exposed and unexposed groups. Multivariate survival analyses were used to control for potential confounders using the ‘streg’ function in STATA. The ‘cluster’ option in STATA was used to allow for the effect of correlations within matched groups on estimates of standard errors and significance levels. Conditional logistic regression analysis was used to explore associations between cases (asthma and depression), controls (asthma but no depression) and to take into account the effect of potential confounders. Standardised mortality ratios (SMR) controlling for age and sex were calculated indirectly.

Sensitivity analyses were conducted to explore the possibility of misclassification of depression and asthma. For depression, the results were re-analysed excluding patients who had also received a Read/OXMIS diagnosis of anxiety or an anxiety disorder, and also by defining depression as a Read/OXMIS code for depression and being prescribed an antidepressant medication. The analyses were also repeated excluding patients who had also received a diagnosis of chronic obstructive pulmonary disease (COPD), and also restricting the inclusion criteria to those aged less than 40 years, as a diagnosis of COPD is unlikely before this age.

#### Ethical Approval

This is an analysis of an anonymised data set. Ethical approval was covered under the terms and conditions of use of the General Practice Research Database via the Medical Research Council.

## Results

### Incidence of depression in patients with asthma

11,275 incident cases of asthma were identified between 1^st^ January 1995 and 31^st^ December 1996 from 219 practices. It was possible to age- sex- and practice-match all cases (ratio 1∶1) giving a total case-control cohort population of 22,550. Fifty seven percent were female. The average age of men was 50 years (s.d.18.9), women 48 years (standard deviation (s.d.) 19) (*p*<0.001). Patients with asthma were followed up in the GRPD for 78,096 person years and patients without asthma for 98,229 person years.

In the population with asthma 1752 were diagnosed with depression over the study period and in the population without asthma, 1353 were diagnosed with depression over the same period.

The incident rate for depression in patients with asthma was 22.4 per 1000 person years. The incidence rate for depression in age-, sex- and practice matched patients without asthma was 13.8 per 1000 person years, giving an incident rate ratio (IRR) of 1.63 (95% CI: 1.52–1.75). There was no statistically significant difference between the incidence rate ratios for depression in the asthmatic population versus non-asthmatic population by sex (IRR men = 1.64 ;95% CI: 1.44–1.87; IRR women 1.63; 95% CI: 1.49–1.77) (Mantel-Haenszel test for homogeneity χ^2^ = 0.01; d.f. 1; *p* = 0.912) or by age (Mantel-Haenszel test for homogeneity χ^2^ = 8.54; d.f. 6; *p* = 0.20). After adjusting, diabetes, cardiovascular disease, cerebrovascular disease, and smoking status the age sex and practice matched IRR was 1.59 (95%CI: 1.48–1.71). Sensitivity analyses did not statistically significantly change the model (sensitivity analyses results available on request from the authors).

### Nested case-control study

Of the 1752 patients with asthma who became depressed, it was possible to match 1660 controls by date of asthma diagnosis (±1 month) giving a total population for the case-control study of 3320 patients. Data on service use were available for 1648 (99%) controls and 1355 (82%) cases. Fifty-three per cent of the controls were women and 70% of the cases. The average age of controls was 49.6 years ( s.d.18.5) and the average age of the cases was 46.1 years (s.d. 18.7; p<0.001). The crude odds ratio for depression in women with asthma compared to men with asthma was 2.09 (95%CI:1.93–2.24). There was a small decreasing trend in the effect of age on depression (crude OR for each additional year 0.99; 95%CI: 0.987–0.994).

The mean number of GP consultations in the year prior to diagnosis of depression in the cases was 8.3 (s.d.7.1) and in the same year in the controls was 5.3 (s.d.5.67) (Mann-Whitney test p<0.001). The age and sex adjusted OR for the association between each GP visit and diagnosis of depression was 1.1 (95% CI 1.07–1.10). The average number of consultations across cases and controls was 6.7 (s.d.6.5). The association between the number of GP visits, severity of asthma and diagnosis of depression is shown in Table 1. The Spearman's correlation coefficient (r) for asthma severity and number of GP visits was 0.3.

**Table 1:** Association between predictor variables and depression in primary care patients with asthma.

	OR^1^	*p*-value	95% Confidence Interval
*Number of GP visits* * *:*			
0–4 visits	1		
5–9 visits	1.85	<0.001	1.53–2.25
10–19 visits	3.21	<0.001	2.53–4.08
≥20 visits	4.51	<0.001	3.20–6.36
Per visit	1.09	<0.001	1.07–1.11
*Asthma severity* ** *:*			
No medication	1		
β2-Agonists	1.26	0.083	0.97–1.64
Inhaled corticosteroids	1.16	0.217	0.92–1.47
Oral corticosteroids	1.76	<0.001	1.33–2.31

1 Adjusted for age and sex.

*In year prior to inclusion into the case-control study (year prior to diagnosis of depression in the cases); [cases n = 1355, controls n = 1647].

**Highest ‘level’ of treatment in year prior to inclusion into the case-control study (year prior to diagnosis of depression in the cases) [cases n = 1660, controls n = 1660].

There were statistically significantly more cases than controls being treated with any anti-asthmatic medication (controls 48.2%, cases 51.8%; *p* = 0.01) and with oral corticosteroids in the year before inclusion in the study (controls 42.6%, cases 57.4%; *p*<0.001). Cases and controls were as likely to have β2 agonists as the highest level of treatment (controls 47.6%, cases 52,4%; *p* = 0.268), and inhaled corticosteroids (controls 51.2%, cases 48.8%; *p* = 0.345). However being treated with an oral corticosteroid was no longer statistically significant after adjusting for the number of GP consultations (See Table 2).

**Table 2:** Adjusted Odds ratios (OR) for depression in primary care patients with asthma.

	Adjusted OR	*p*-value	95% confidence interval
*GP visits in year prior to study inclusion* (cases n = 1355, controls n = 1647):			
<5 visits	1		
5–9 visits	1.91*	<0.001	1.57–2.31
10–19 visits	3.35*	<0.001	2.62–4.28
≥20 visits	4.70*	<0.001	3.32–6.65
Per visit	1.09*	<0.001	1.07–1.11
*Asthma severity* (cases n = 1660, controls n = 1660):			
No medication	1		
β2-Agonists	0.83**	0.192	0.64–1.09
Inhaled corticosteroids	0.81**	0.056	0.66–1.01
Oral corticosteroids	0.88**	0.372	0.67–1.16
*SMR* 1	1.87		1.54–2.27

*Adjusted for age, sex and severity (type of treatment in the year prior to inclusion in the study).

**Adjusted for age, sex and number of GP visits in year prior to inclusion in study.

1 Age and Sex Standardised Mortality Ratio for primary care patients with asthma and depression versus those with asthma but no depression.

The age and sex standardised SMR for primary care patients with asthma compared to the general primary care population was 2.86 (95%CI: 2.64–3.10). The age and sex standardised mortality ratio for primary care patients with asthma and depression compared with non-depressed patients with asthma was 1.87 (95%CI: 1.54–2.27).This did not change statistically significantly with asthma severity.

## Discussion

We have found a statistically significant association between a diagnosis of asthma and the incidence of depression in primary care patients, and a higher mortality rate in patients with asthma and depression compared to patients with asthma only. This finding is particularly important as asthma is one of the commonest chronic conditions treated in primary care and an association between asthma and depression has potentially important implications for public health. In the UK, case-finding for depression has been instituted for primary care patients with diabetes and coronary heart disease [21], but not for asthma. Asthma is more common than coronary heart disease [22], [23] and diabetes [24], [23]. Our results suggest the association between depression and asthma may be as strong as that between depression in coronary heart disease and diabetes. Case-finding for depression in this population should therefore be considered.

An increased mortality rate in patients with co-morbid asthma and depression has also been reported for asthma patients treated in tertiary care [4]. It has been hypothesised that concurrent depression leads to poor adherence with asthma treatments and hence to poorer outcomes [25]. Eisner *et al* reported that depressive symptoms were also associated with an increased risk of being hospitalised for asthma [26]. Due to limitations of the dataset we were unable to determine the cause of death in our study and further research is needed to explore the reasons for the increased mortality in this population.

Our results suggest that asthma predisposes to developing depression. However, this did not appear to be contingent on asthma severity. Evidence for an association between asthma severity and depression is contradictory. Eisner *et al* found that in a prospective cohort of patients hospitalised for asthma, depressive symptoms did increase with asthma severity [26], though Chapman *et al* reported that patients with mild asthma reported depression as frequently as those with severe asthma [27]. Our results are in line with those of Chapman *et al*. The reason for this counter-intuitive result is unclear. One explanation is that objective measures of asthma severity (such as peak expiratory flow rate) do not necessarily reflect subjective experience [14].

Oral corticosteroid use is unlikely to be the cause of the increased rate of depression in patients with asthma. We failed to find an association between inhaled or oral corticosteroid use and depression after adjusting for number of primary care consultations. The correlation between asthma severity and number of primary care consultations was low. Frequent attendance in primary care has been associated with receiving a diagnosis of depression and so patients with asthma who are frequent attendees may have a higher likelihood of being diagnosed with depression. An increased frequency of attendance may increase the opportunity for primary care doctors to detect depression or may reflect an underlying depression leading to an increased number of consultations. Primary care physicians should therefore consider depression in frequent attendees who have asthma.

### Limitations

The data in the GPRD are essentially computerised general practice records, completed as part of routine clinical care. They should therefore reflect clinical practice. However there are limitations. Diagnoses were those given by GPs and no standardised instruments were used to confirm this diagnosis. There have been no studies we are aware of that have examined the validity of diagnoses for depression in the GPRD, though a study of psychosis diagnoses using this database found high predictive values [28]. We conducted sensitivity analyses to explore the possible effects of misclassification of depression and these were minimal. Loss to follow-up from the cohort should also be minimal as registration with primary care and exits from the database are carefully recorded. We were unable to assess the severity of asthma directly, and had to use a medication based proxy, though this has been used successfully in other studies [20]. However we were unable to measure adherence to medications. Poor adherence may result in fewer visits to a GP because patients would not have to attend to for further prescriptions, or it could result in poorer asthma control which may in turn result in more visits. Poor compliance may therefore be a residual confounder.

The case-control study may be subject to residual confounding for example from socioeconomic status. The relationship between socioeconomic status and asthma is unclear with conflicting evidence of an association [29]. Likewise, though there appears to be a relationship between the prognosis of depression and socioeconomic status, the association between the incidence of depression and socioeconomic status is less clear [30]. Nevertheless we considered socioeconomic status to be a potential confounder. We were unable to assess socioeconomic status directly and had to use GP practice as a proxy. GP practice has been found to correlate with socioeconomic status at the individual level, though it is only an approximation of the area-based deprivation of the practice population as a whole [19]. This may lead the strength of the association between asthma and depression to be overestimated. It is unlikely that frequency of attendance at GP practices is confounded by socioeconomic status. Higher rates of consultation have not been found to be related to socioeconomic status, but rather the number of underlying chronic medical conditions (which is associated to socioeconomic status) [31]. We controlled for most of the common chronic medical conditions in general practice and though there may still be residual confounding from medical conditions we did not control for this is likely small.

In conclusion, there is a higher incidence of depression recorded by GPs in patients with asthma, and co-morbid asthma and depression increases mortality. A diagnosis of depression does not seem to be related to asthma severity, but is related to the frequency of GP consultation. Depression screening should be considered in primary care patients with asthma.

## References

[1] Health and Social Care Information Centre (2005). National Quality and Outcomes framework statistics for England 2004/5..

[2] Ustun TB, Sartorius N (1995). Mental illness in general health care. An international study.

[3] Walters P, Ashworth M, Tylee A (2008). Ethnic density, physical illness, social deprivation and antidepressant prescribing in primary care: ecological study.. British Journal of Psychiatry.

[4] Mancuso CA, Peterson MG, Charlson ME (2000). Effects of depressive symptoms on health-related quality of life in asthma patients.. Journal of General Internal Medicine.

[5] Nascimento I, Nardi AE, Valenca AM, Lopes FL, Mezzasalma MA (2002). Psychiatric disorders in asthmatic outpatients.. Psychiatry Research.

[6] Goldney RD, Ruffin R, Fisher LJ, Wilson DH (2003). Asthma symptoms associated with depression and lower quality of life: a population survey.. Medical Journal of Australia.

[7] Adams RJ, Wilson DH, Taylor AW, Daly A, Tursan DE (2004). Psychological factors and asthma quality of life: a population based study.. Thorax.

[8] Chapman DP, Perry GS, Strine TW (2005). The vital link between chronic disease and depressive disorders.. Preventing Chronic Disease.

[9] Ng TP, Chiam PC, Kua EH (2007). Mental disorders and asthma in the elderly: a population-based study.. International Journal of Geriatric Psychiatry.

[10] Scott KM, Bruffaerts R, Tsang A, Ormel J, Alonso J (2007). Depression-anxiety relationships with chronic physical conditions: results from the World Mental Health Surveys.. Journal of Affective Disorders.

[11] Scott KM, Von Korff M, Ormel J, Zhang MY, Bruffaerts R (2007). Mental disorders among adults with asthma: results from the World Mental Health Survey.. General Hospital Psychiatry.

[12] Goodwin RD, Jacobi F, Thefeld W (2003). Mental disorders and asthma in the community.. Archives of General Psychiatry.

[13] Goodwin RD, Fergusson DM, Horwood LJ (2004). Asthma and depressive and anxiety disorders among young persons in the community.. Psychological Medicine.

[14] Opolski M, Wilson I (2005). Asthma and depression: a pragmatic review of the literature and recommendations for future research.. Clinical Practice and Epidemiology in Mental Health.

[15] Perry J (1978). OXMIS problem codes for primary medical care.

[16] Hansell A, Hollowell J, Nichols T, McNiece R, Strachan D (1999). Use of the General Practice Research Database (GPRD) for respiratory epidemiology: a comparison with the 4th Morbidity Survey in General Practice (MSGP4).. Thorax.

[17] Anderson HR, Steven A, Raferty J (1994). Lower respiratory disease.. Health Needs Assessment: the epidemiologically based needs assessment reviews.

[18] Rodriguez LAG, Gutthann SP (1998). Use of the UK General Practice Research Database for pharmacoepidemiology.. Br J Clin Pharmacol.

[19] Strong M, Maheswaran R, Pearson T (2006). A comparison of methods for calculating general practice level socioeconomic deprivation.. International Journal of Health Geographics.

[20] Tata L J, Lewis SA, McKeever TM, Smith CJ, Doyle P (2007). A comprehensive analysis of adverse obstetric and pediatric complications in women with asthma.. American Journal of Respiratory & Critical Care Medicine.

[21] The Information Centre for Health and Social Care (2007). QOF.. http://www.ic.nhs.uk/statistics-and-data-collections/audits-and-performance/the-quality-and-outcomes-framework.

[22] Rudisch B, Nemeroff CB (2003). Epidemiology of comorbid coronary artery disease and depression.. Biological Psychiatry.

[23] Evans DL, Charney DS, Lewis L, Golden RN, Gorman JM (2005). Mood disorders in the medically ill: scientific review and recommendations.. Biological Psychiatry.

[24] Anderson RJ, Freedland KE, Clouse RE, Lustman PJ (2001). The prevalence of comorbid depression in adults with diabetes: a meta-analysis.. Diabetes Care.

[25] Cluley S, Cochrane GM (2001). Psychological disorder in asthma is associated with poor control and poor adherence to inhaled steroids.. Respiratory Medicine.

[26] Eisner MD, Katz PP, Lactao G (2005). Impact of depressive symptoms on adult asthma outcomes.. Ann Allergy Asthma Immunol.

[27] Chapman KR (2005). Impact of ‘mild’ asthma on health outcomes: findings of a systematic search of the literature.. Respiratory Medicine.

[28] Nazareth I, King M, Haines A, Tai SS, Hall G (1993). Care of schizophrenia in general practice.. BMJ.

[29] Hancox RJ, Milne BJ, Taylor DR, Greene JM, Cowan JO (2004). Relationship between socioeconomic status and asthma: a longitudinal cohort study.. Thorax.

[30] Weich S, Churchill R, Lewis G, Mann A (1997). Do socio-economic risk factors predict the incidence and maintenance of psychiatric disorder in primary care?. Psychological Medicine.

[31] Wyke S, Hunt K, Walker J, Wilson P (2003). Frequent attendance, socioeconomic status and burden of ill health. An investigation in the west of Scotland.. European Journal of General Practice.

